# Down-regulation of human topoisomerase IIα expression correlates with relative amounts of specificity factors Sp1 and Sp3 bound at proximal and distal promoter regions

**DOI:** 10.1186/1471-2199-8-36

**Published:** 2007-05-20

**Authors:** Amram O Williams, Richard J Isaacs, Kathryn M Stowell

**Affiliations:** 1 Institute of Molecular Biosciences, Massey University, Private Bag 11-222, Palmerston North, New Zealand; 2 Regional Cancer Treatment Service, Palmerston North Public Hospital, Ruahine Street, Palmerston North, New Zealand

## Abstract

**Background:**

Topoisomerase IIα has been shown to be down-regulated in doxorubicin-resistant cell lines. The specificity proteins Sp1 and Sp3 have been implicated in regulation of topoisomerase IIα transcription, although the mechanism by which they regulate expression is not fully understood. Sp1 has been shown to bind specifically to both proximal and distal GC elements of the human topoisomerase IIα promoter *in vitro*, while Sp3 binds only to the distal GC element unless additional flanking sequences are included. While Sp1 is thought to be an activator of human topoisomerase IIα, the functional significance of Sp3 binding is not known. Therefore, we sought to determine the functional relationship between Sp1 and Sp3 binding to the topoisomerase IIα promoter *in vivo*. We investigated endogenous levels of Sp1, Sp3 and topoisomerase IIα as well as binding of both Sp1 and Sp3 to the GC boxes of the topoisomerase IIα promoter in breast cancer cell lines *in vivo *after short term doxorubicin exposure.

**Results:**

Functional effects of Sp1 and Sp3 were studied using transient cotransfection assays using a topoisomerase IIα promoter reporter construct. The *in vivo *interactions of Sp1 and Sp3 with the GC elements of the topoisomerase IIα promoter were studied in doxorubicin-treated breast cancer cell lines using chromatin immunoprecipitation assays. Relative amounts of endogenous proteins were measured using immunoblotting. *In vivo *DNA looping mediated by proteins bound at the GC1 and GC2 elements was studied using the chromatin conformation capture assay. Both Sp1 and Sp3 bound to the GC1 and GC2 regions. Sp1 and Sp3 were transcriptional activators and repressors respectively, with Sp3 repression being dominant over Sp1-mediated activation. The GC1 and GC2 elements are linked *in vivo *to form a loop, thus bringing distal regulatory elements and their cognate transcription factors into close proximity with the transcription start site.

**Conclusion:**

These observations provide a mechanistic explanation for the modulation of topoisomerase IIα and concomitant down-regulation that can be mediated by topoisomerase II poisons. Competition between Sp1 and Sp3 for the same cognate DNA would result in activation or repression depending on absolute amounts of each transcription factor in cells treated with doxorubicin.

## Background

Topoisomerases are ubiquitous enzymes that alter DNA topology by cleavage and religation of the DNA. Human topoisomerase II exists as two isoforms, known as topoisomerase IIα (174 kDa) and topoisomerase IIβ (182 kDa), which are essential for a number of nuclear processes, including chromosome condensation, chromatid separation, and relief of torsional stress during DNA replication and transcription [1]. These topoisomerases have been shown to be targeted by common anti-cancer drugs known as topoisomerase poisons, including acridines, anthracyclines, and actinomycins as they stabilize the enzyme-mediated double-stranded breaks in the DNA [2]. Drug sensitivity to the topoisomerase II poisons is dependent on high levels of topoisomerase II in addition to the presence of the drug, while drug resistance has been correlated with low levels of the enzyme. Doxorubicin has been shown to be most effective when cells are rapidly proliferating and expressing high levels of topoisomerase IIα, the critical nuclear enzyme in maintaining correct DNA topology, which is vital for cell function and survival. Many factors have been implicated in the development of resistance to chemotherapeutic drugs but the down-regulation of topoisomerase II is believed to be an important mechanism underlying the acquired drug resistance [3-5]. The molecular mechanisms linking drug treatment and down-regulation of topoisomerase II expression are incompletely understood. For example it is not known whether the down-regulation of topoisomerase II expression is the result of early changes in expression of key transcription factors or more subtle effects that occur over a longer time course.

The human topoisomerase IIα promoter has five functional CCAAT boxes (ICB1-5) and two GC boxes [6]. CCAAT boxes are customarily flanked by at least one functionally important promoter element. In the case of the human topoisomerase IIα promoter ICB1 and ICB5 are flanked by GC1 and GC2 respectively. GC boxes are the second most frequent element in promoters and tend to be present in multiple copies. In promoters where multiple GC boxes are present, it is common for each GC box to have a different function according to its position with respect to the transcription start point and other flanking elements.

Within the human topoisomerase IIα promoter, GC1 is contained in the minimal promoter [6], whereas GC2 is distal to this region. Mutations in GC1 have shown no marked decrease in topoisomerase IIα activity, however in resistant cells a mutation in GC1 has been shown to increase promoter activity (reviewed in [7]).

Although some promoters have multiple GC boxes, it has been shown that a single Sp1 binding site is sufficient to stimulate promoter activity [8]. Sp1 is commonly known as a transcriptional activator and has been shown to be able to up-regulate transcription in a variety of promoters including topoisomerase IIα [9]. Sp1-mediated transcriptional activation depends on 3 zinc finger structures responsible for DNA binding, and at least one of two Q-rich regions (or activation motifs) that are required for protein/protein interactions and coincident synergistic transactivation [10].

Sp3 is similar to Sp1 in a number of ways: it is ubiquitously expressed in mammalian cells, has increased DNA binding affinity when phosphorylated, and has the ability to compete with the same target sequences with similar binding affinities due to similar DNA binding domains. The major difference between the two transcription factors is that Sp3 is bifunctional, possessing the ability to act not only as a repressor, but also as an activator. The repressive function of Sp3 has been attributed to a region near the N-terminus, which acts as a transcriptional repressor of several activators. It is thought that promoters with a single binding site can be activated by Sp3, but those with multiple binding sites respond weakly to Sp3 or not at all [11]. While Sp3 may be able to form homo-oligomers synergistic transactivation does not occur [12].

Important chromosomal activities and the properties of local chromatin structure affect gene expression, origin firing, and DNA replication [13]. First described by Dekker *et al*., 2002 [14], chromosome conformation capture allows the analysis of spatial organization of chromosomes. This method affords high resolution and specific definition of chromatin conformation. DNA looping has been suggested in earlier reports [9,15] to have important implications in the functional properties of protein-promoter interactions particularly in the case of Sp1/Sp3 interactions with the GC1 and GC2 regions in the human topoisomerase IIα promoter.

In this study we explore the regulation of the human topoisomerase IIα promoter *in vivo *using MDA MB 231 cells in the early stages of treatment with doxorubicin, a cytotoxic drug commonly used to treat breast cancer. We show that Sp3 has a dominant repressive effect on Sp1-mediated activation. With lower levels of topoisomerase IIα evident in drug treated breast cancer cells, these data suggest that Sp3 may play a vital role in the development of resistance to chemotherapy and supports earlier work that suggested Sp3 was upregulated in etoposide/teniposide-resistant KB cell lines [16]. We also show that GC1 and GC2 are indeed on a DNA loop indicating that both GC regions play an important part in the regulation of the promoter.

## Results

### Transcription factors Sp1 and Sp3 bind to both GC1 and GC2

**Figure 1:**
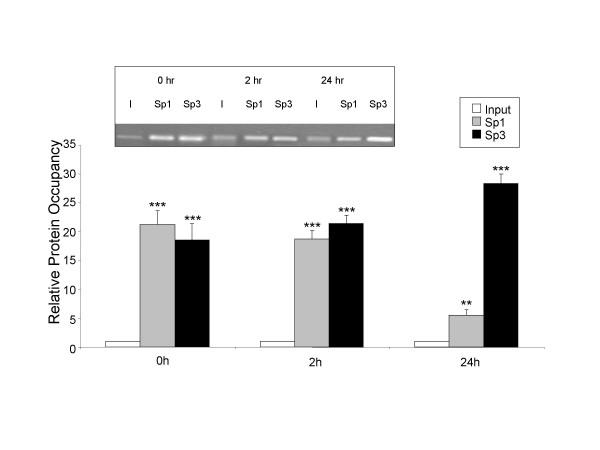
Occupancy of Sp1 at GC1 of the topoisomerase IIα promoter decreases after doxorubicin treatment. ChIP analysis was carried out using MDA MB 231 breast cancer cells. Cells were exposed to a single dose of doxorubicin (5 μM) for 1 hour and cell extracts were taken at 0 hr (control), 2 hr and 24 hr after initial exposure. Each value represents the average and standard error of the fold enrichment in three experiments carried out in triplicate. Fold enrichment values are "relative protein occupancies" calculated by subtracting background levels from observed immunoprecipitation efficiencies. The corresponding gel photograph (inset) shows PCR products from real time PCR of Sp1 or Sp3 binding to GC1. in unexposed (0 hr) and exposed (2 h and 24 h) cells. I indicates input control. Results are expressed as ± SEM where n = 9, *** p < 0.001, ** p < 0.01, and * p < 0.05.

**Figure 2:**
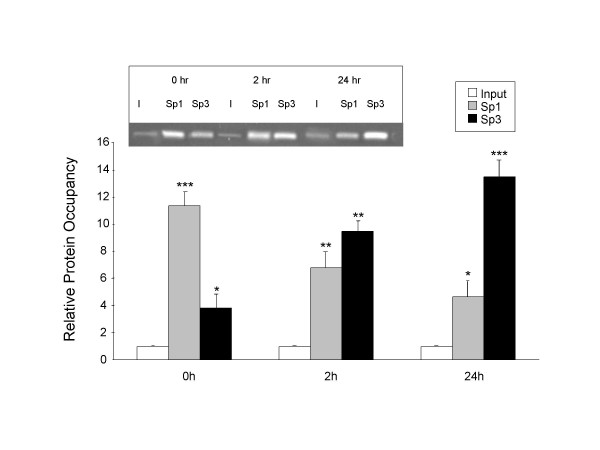
Occupancy of Sp3 at GC2 of the topoisomerase IIα promoter increases after doxorubicin treatment. ChIP analysis was carried out using MDA MB 231 breast cancer cells. Cells were exposed to a single dose of doxorubicin (5 μM) for 1 hour and cell extracts were taken at 0 hr (control), 2 hr and 24 hr after initial exposure. Values were calculated as in the legend for Figure 1 and represent the average and standard error of the fold enrichment in three experiments carried out in triplicate. The corresponding gel photograph (inset) shows PCR products from real time PCR of Sp1 or Sp3 binding to GC2 in unexposed (0 hr) and exposed (2 h and 24 h) cells. I indicates input control. Results are expressed as ± SEM where n = 9, *** p < 0.001, ** p < 0.01, and * p < 0.05.

Chromatin immunoprecipitation analysis using MDA MB 231 cells shows that both the GC1 and GC2 elements of the topoisomerase IIα promoter are occupied by both Sp1 and Sp3 *in vivo *(Figures 1 and 2). Fold enrichment values are presented as "relative occupancy units" and were calculated by subtracting background immunoprecipitation efficiencies from observed immunoprecipitation efficiencies. Input was arbitrarily set at 1 after background correction. For GC1 binding, the Sp1 control (0 hour treatment) was found to have 20 occupancy units over the input, while the 2 and 24 hour treatments were shown to have 17 and 4 occupancy units respectively. Association of Sp3 with GC1 at the control (0 hour) and 2 hour treatments is comparable to that of Sp1 association, with 17 and 20 occupancy units respectively. Sp3 data at the 24 hour time point, on the other hand, show higher relative protein occupancy. Sp1 occupancy units at GC2 for control (0 hour), 2 hour, and 24 hour treatments were 11, 7, and 5 respectively. Sp3 protein occupancy at GC2 showed a reverse trend for the same treatment times with 3, 10, and 13 units.

These data suggest that more Sp1 and Sp3 protein can bind to GC1 *in vivo *than to GC2. When exposed to doxorubicin, there is no significant change in occupancy at GC1 for either Sp1 or Sp3 at 0 hour and 2 hours after exposure (insets of Figure 1 and Figure 2). There is however, a significant difference between Sp1 and Sp3 occupancy 24 hours after exposure, with the association between Sp3 and GC1 being 6.75 fold greater than Sp1 association with GC1. The occupancy by Sp1 and Sp3 of GC2, however, is decreased by 20% and 50 % respectively after 2 hours of drug exposure and by 50 % and 100% respectively after 24 hours of drug exposure (Figure 1 and Figure 2). While we cannot rule out the presence of a larger template containing both the GC1 and GC2 elements, these differences in occupancy suggest that separated DNA elements were in fact immunoprecipitated and support the conclusion that both Sp1 and Sp3 bind to each of GC1 and GC2 *in vivo*.

### Sp1 and Sp3 act as transcriptional activator and repressor respectively

**Figure 3:**
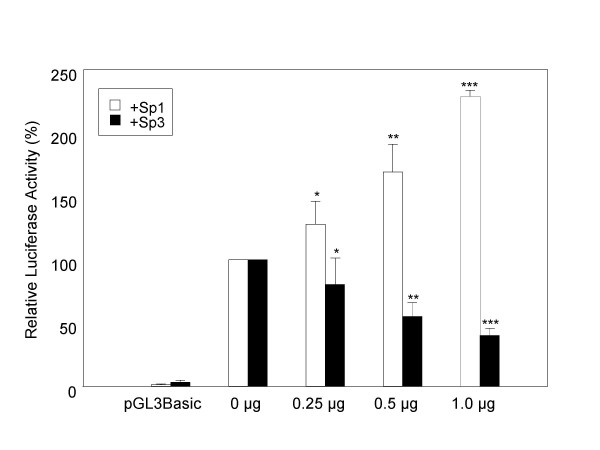
Sp1 and Sp3 are antagonistic at the topoisomerase IIα promoter. All transfections were performed in triplicate with 1.0 μg of -617 wt topoisomerase IIα reporter plasmid and 1 μg of pCMV Sport β-galactosidase control vector. Relative luciferase activity was calculated by arbitrarily setting the control (0 μg, containing only topoisomerase IIα reporter plasmid and pCMV Sport β-galactosidase control vector) to 100%. Results are expressed as ± SEM where n = 9, *** p < 0.001, ** p < 0.01, and * p < 0.05.

MDA MB 231 cells were transiently transfected with the luciferase reporter vector pGL3Basic incorporating the -617 promoter of human topoisomerase IIα [6,17]. This region contains both the distal GC2 element and the proximal GC1 element. In each assay, the pCMV Sport β-galactosidase vector was used as a control for transfection efficiency. The effects of Sp1 and Sp3 on transcription were tested by co-transfecting increasing amounts of Sp1 and Sp3 expression vectors respectively (Figure 3). Sp1 has been shown previously to be a transcriptional activator in both HeLa [9] and MDA MB 231 [18]. Figure 3 clearly shows Sp3 acts as a transcriptional repressor of human topoisomerase IIα by decreasing expression in a dose-dependent manner by approximately 60% at the highest concentration used.

**Figure 4:**
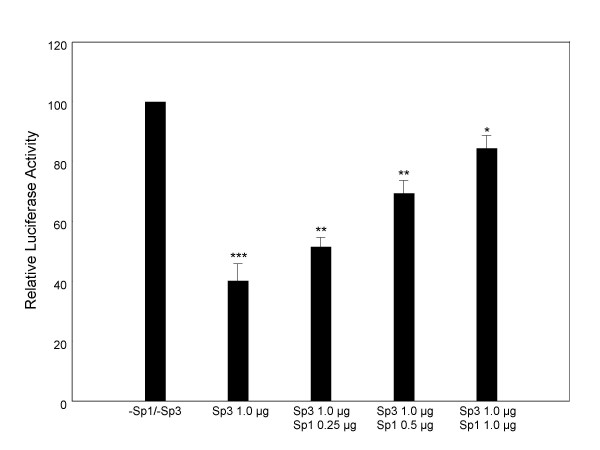
Sp3 maintains transcriptional repression in the presence of Sp1. All transfections were carried out in triplicate with 1.0 μg of -617 wt topoisomerase IIα reporter plasmid and 1 μg of pCMV Sport β-galactosidase control vector. Relative luciferase activity was calculated by arbitrarily setting the control (no added Sp1 or Sp3) to 100%. Results are expressed as ± SEM where n = 9, *** p < 0.001, ** p < 0.01, and * p < 0.05

**Figure 5:**
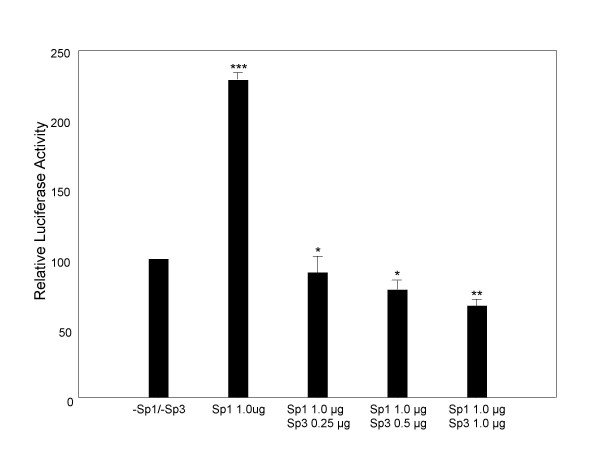
Sp3 transcriptional repression is dominant over activation by Sp1. All transfections were performed in triplicate with 1.0 μg of -617 wt topoisomerase IIα reporter plasmid and 1 μg of pCMV Sport β-galactosidase control vector. Relative luciferase activity was calculated by arbitrarily setting the control (no added Sp1 or Sp3) to 100%. Results are expressed as ± SEM where n = 9, *** p < 0.001, ** p < 0.01, and * p < 0.05

Since Sp1 and Sp3 can bind to both GC elements *in vivo *as shown in figures 1 and 2 we sought to determine whether one had a dominant functional effect over the other. Transient transfections using a range of concentrations of Sp1 and Sp3 were carried out. These experiments show that the repressive effect of Sp3 can only partially be overcome by Sp1 (Figure 4) where with equal quantities of added Sp1 and Sp3, transcriptional activity is approaching control values. In addition, the activating effect of Sp1 is completely abolished by Sp3 where with even a 4:1 ratio of Sp1:Sp3 the transcriptional activity is the same as the control (Figure 5). These results suggest that Sp1 and Sp3 are activators and repressors respectively of human topoisomerase IIα *in vivo*. More importantly they suggest that Sp3 is functionally dominant over Sp1.

### Expression of endogenous topoisomerase II alpha, Sp1 and Sp3 in doxorubicin exposed breast cancer cells

**Figure 6:**
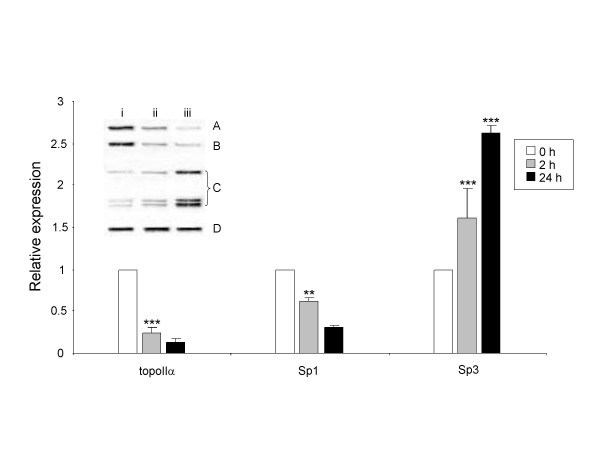
Endogenous topoisomerase IIα decreases concomitantly with decreased Sp1 and increased Sp3 after treatment with doxorubicin. (A) Topoisomerase IIα; (B) Sp1; (C) Sp3; (D) tubulin control, at time points 0 hours (i), 2 hours (ii), and 24 hours (iii) after exposure to doxorubicin in MDA MB 231 cells. The three proteins bracketed in C represent the isoforms of Sp3 produced from alternative start codons. Results show the average of three experiments carried out in triplicate and are expressed as ± SEM where n = 9, *** p < 0.001, ** p < 0.01, and * p < 0.05. The insert shows one indicative immunoblot.

Semi-quantitative immunoblotting of cell extracts was carried out and the amounts of topoisomerase IIα, Sp1, and Sp3 were expressed as a percentage relative to tubulin and normalised to the control (0 hr) which was arbitrarily set at 100%. The results showed decreased amounts of topoisomerase IIα and Sp1 at 2 and 24 hours after exposure when compared to unexposed (0 hr) control cells. Amount of Sp3 increased in relation to the control (Figure 6). These results clearly show a significant difference between the control and drug-exposed cells for all proteins at each time point and suggest that the changes in expression of Sp1, Sp3, and topoisomerase IIα are the result of exposure to doxorubicin. The molecular mechanism responsible for the altered expression of both Sp1 and Sp3, however, is uncertain.

### GC1 and GC2 exist in close proximity on looped DNA in HeLa cells

**Figure 7:**
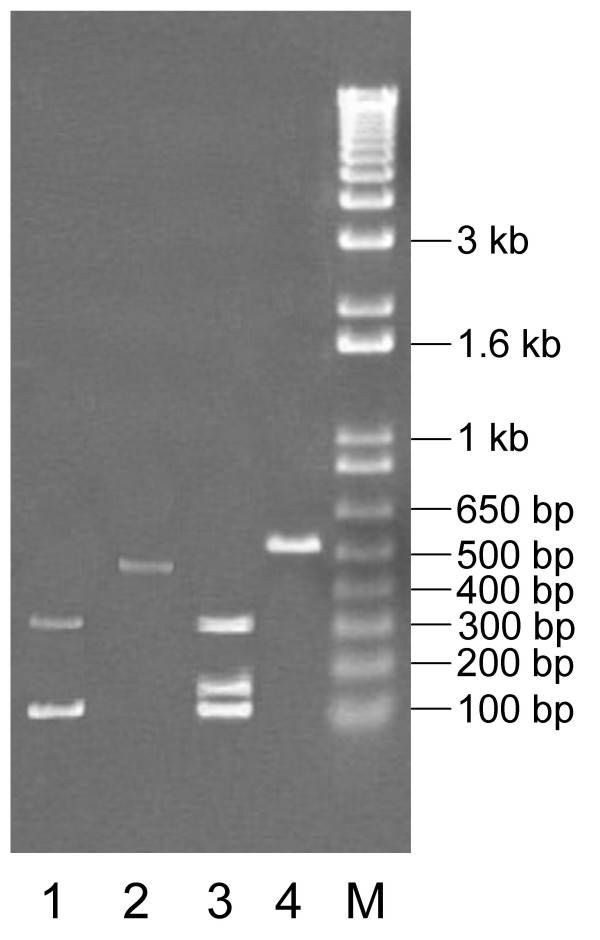
The proximal topoisomerase IIα promoter forms a loop between GC1 and GC2. A ligation product (429 bp) was detected by PCR (lane 2) after completion of the 3C assay. Digestion of ligated product (lane 1) with *BSu*36 I yielded two fragments (123 bp and 306 bp). The PCR product from the uncut genomic DNA control (lane 4) was 571 bp in size. When digested with *BSu*36 I, genomic DNA product (lane 3) yielded three fragments (123, 306 and 142 bp) as predicted from the DNA sequence.

Chromosome conformation capture results (Figure 7) show that GC1 and GC2 are located within close spatial proximity on the human topoisomerase IIα promoter *in vivo *as suggested previously [9,15]. The results show an uncut PCR product representing ligated promoter in lane 2, with a size of 429 bp. This is in comparison to the uncut PCR product produced from untreated genomic DNA in lane 4 (571 bp). Digestion of the ligation product (lane 1) yields two fragments (123 and 306 bp), while digestion of the genomic DNA product (lane 3) yields three fragments (123, 142, and 306 bp) as predicted from the genomic DNA sequence. These results suggest that the GC1 and GC2 elements are normally (in the absence of drug) positioned *in vivo *close to the transcription start point by virtue of the interactions between Sp1 and/or other factors with the cognate DNA.

## Discussion

While it has been documented that Sp1 binds to GC rich regions in the topoisomerase IIα promoter [9] there has been no study reported to date that addresses Specificity protein/GC interactions at the topoisomerase IIα promoter *in vivo*. Here we show, for the first time, that both Sp1 and Sp3 bind to both the GC1 and GC2 elements of the topoisomerase IIα promoter *in vivo *and there is a greater occupancy of both proteins at GC1 than at GC2. These observations implicate GC1 as having a major role in basal transcription of topoisomerase IIα while GC2 may function in a more modulatory capacity. We have previously shown that Sp1 acting at the GC2 element may repress basal transcription via a putative interaction at the ICB1 and GC1 elements either by direct interaction or the recruitment of other regulatory proteins [9]. These findings suggested that proteins bound to the GC2 element may act as repressors, which is supported by the current data from ChIP analysis indicating that both Sp1 and Sp3 can interact *in vivo *with GC2 (Figures 1 and 2). Putative interactions with other transcriptional modulators however, cannot be ruled out. Chromosome conformation capture assays used in this study support the findings suggested by Mastrangelo *et al., *1991 [15], that there is a physical loop formed which results in a strongly increased concentration of transcriptional modulators at the proximal promoter. It has been reported that transcription activities are related to the overall spatial arrangements between protein transcription factors and their cognate DNA elements [19-22]. We show that Sp1 and Sp3 bind to both GC1 and GC2 *in vivo *and are likely to modulate transcription due to both DNA/protein (Sp1 and/or Sp3) interactions and protein/protein interactions between Sp1 facilitated by DNA looping between the GC1 and GC2 elements of the topoisomerase IIα promoter. As Sp3 does not form multimers or interact with Sp1 [12], the repressive effect of Sp3 is likely to be due to its dominant effect over Sp1. Our data suggest that Sp3 can displace Sp1 from either or both GC1 and GC2 and this would be likely to open the chromatin loop.

We have previously shown that Sp1 can up-regulate transcription of topoisomerase IIα [9,18] and this current study supports Sp1 as a transcriptional activator of topoisomerase IIα. These findings are consistent with other work, which has shown that in the rat topoisomerase IIα promoter, Sp1 is a transcriptional activator at the GC2 element, which is spatially equivalent to the GC1 element of the human topoisomerase IIα promoter [23].

More importantly we show for the first time that Sp3 is a transcriptional repressor of topoisomerase IIα that is functionally dominant over Sp1. Immunoblotting results indicate that changes in expression levels of these proteins occur with exposure to chemotherapeutic agents. Other reports suggest that in some promoter contexts both Sp1 and Sp3 have both activator [24-27] and repressor functions [28,29]. Moreover, the repressive functions of Sp3 transcription factors have recently been implicated in breast cancer cell lines [30,31]. Reduced Sp3 RNA levels in merbarone-resistant human leukemic CEM cells have been shown to correlate with a down-regulation of topoisomerase IIα protein levels [32]. These authors also showed that co-transfection of Sp3 induced topoisomerase IIα promoter activity in drug sensitive CEM cells in a dose dependent manner. Conversely, Sp3 levels were shown to be elevated in etoposide/teniposide-resistant KB cells, and this elevation correlated with a decrease in topoisomerase IIα activity [16]. Taken together, these observations suggest that Sp3 is normally a transcriptional repressor of topoisomerase IIα, but its effects are likely to be cell type specific.

In co-transfection studies using Sp1 and/or Sp3 expression plasmids other reports have shown that while Sp1 stimulated, Sp3 repressed Sp1-mediated transactivation of human transcobalanin II (TC II) transcription [33]. In functional analyses using Drosophila SL-2 cells, Sp1 expression can drive transcription from the elastin proximal promoter, while co-expression of Sp3 results in a repression of Sp1 activity [34]. Functional assays have suggested that TGF-beta1 inhibition of COL2A1 gene transcription in rabbit articular chondrocytes is mediated by an increase of the Sp3/Sp1 ratio and by the repression of Sp1 transactivating effects on that gene [35]. These authors found that both proteins bind to the same sites of the c-myc promoter. Co-transfection experiments, in mammalian and insect cells, indicated that Sp1 trans-activated the c-myc promoter, whereas Sp3 did not. In addition, enforced expression of Sp3 repressed Sp1-mediated activation of c-myc [11]. These data support our findings for the transcriptional regulation of human topoisomerase IIα and suggest that Sp3 may be a common modulator of Sp1-dependent transcriptional activation.

The dominant repressive activity of Sp3 over Sp1 has important implications for the expression of topoisomerase IIα in doxorubicin-based chemotherapy. Our results show that in unexposed and drug exposed cells, Sp1 and Sp3 both interact with the topoisomerase IIα promoter at the GC1 and GC2 elements *in vivo*. We have previously shown an interaction between both Sp1 and Sp3 with an isolated GC2 element *in vitro *but Sp3 was only able to bind to a composite element containing both ICB1 and GC1 [9]. This suggests more complex interactions may occur between DNA and protein *in vivo *and conformation of the chromatin in the topoisomerase IIα promoter may have a critical role in allowing access and binding of transcription factors. In addition, while both Sp1 and Sp3 are able to interact with GC2 in untreated cells, occupancy at this site is significantly less than at GC1. We, and others have shown that topoisomerase IIα expression levels are reduced in drug resistant breast cancer cells. Co-transfection assays have shown that Sp3 negatively impacts Sp1-mediated activation. Sp1/Sp3 co-transfection results, coupled with the results of Sp3 ChIP assays therefore suggest that Sp3 plays a critical role in the down-regulation of topoisomerase IIα in drug treated breast cancer cells. We have previously shown that the absolute amounts of Sp3 in some doxorubicin-resistant cell lines with reduced topoisomerase IIα did not alter significantly, while Sp1 levels were significantly reduced [9]. Taken together these data support the suggestion that the ratio of Sp1/Sp3 is likely to be a critical factor in determining the level of expression of topoisomerase IIα and hence sensitivity to doxorubicin treatment.

**Figure 8:**
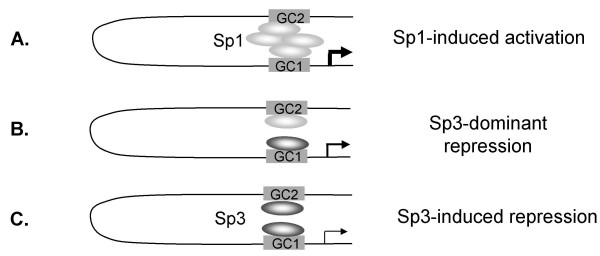
Transcriptional modulation of topoisomerase IIα facilitated by DNA looping. (A) High levels of Sp1 and the formation of Sp1 multimers results in increased transcriptional activity of the topoisomerase IIα promoter. (B) A repressive effect on promoter activity when Sp1/3 levels are equal as Sp1 can no longer form protein/protein interactions because of Sp3 occupancy at either GC1 or GC2. (C) Higher Sp3 levels show a dominant repressive effect of promoter activity as most GC1 and GC2 elements are occupied by Sp3.

We have previously suggested that DNA looping at the topoisomerase IIα promoter may be involved in determining expression levels of the gene [9,36]. The results presented here demonstrate that a DNA loop can be formed at the topoisomerase IIα promoter between the GC1 and GC2 elements. When Sp1 levels are sufficiently high, it is likely that occupancy at both GC1 and GC2 occurs to form a multi-protein/DNA complex and maximal transcription. Drug treatment reduces Sp1 levels and subsequently causes a reduction in binding at both GC1 and GC2. At the same time Sp3 levels rise and while occupancy at GC1 does not change markedly, there is a significant increase of occupancy at GC2 by Sp3. Sp3, in preference to Sp1, bound at both GC1 and GC2 would cause opening of the chromatin loop (because Sp3 does not self-associate as does Sp1) and a concomitant decrease in topoisomerase IIα expression. This correlates with the up-regulation of expression we observed previously in transient transfection assays using the -617 topoisomerase IIα promoter carrying a point mutation in the GC2 element, that would preclude binding of Sp3 [9]. Where ratios of Sp1/Sp3 are high a multiprotein/DNA complex is likely to form, with accompanying high levels of expression (Figure 8A). As the ratio of Sp1/Sp3 drops with drug treatment Sp3 will start to replace Sp1 at both GC1 and GC2 with less likelihood of forming a chromatin loop (Figure 8B and 8C). Absolute levels of transcription will then depend upon the ratio of Sp1/Sp3 in the cell but as Sp3 is dominant over Sp1, relatively small increases in Sp3 levels or decreases in Sp1/Sp3 ratio could result in reduced transcription of topoisomerase IIα. As these effects occur early in drug treatment, the expression levels of Sp1 and Sp3 may be the primary determinants of topoisomerase IIα expression in the early stages of chemotherapy. Initial low protein levels of topoisomerase IIα as shown in Figure 6, would be predicted to result in fewer cleavable complexes allowing effective DNA repair to take place, consequently leading to drug resistance in the longer term.

## Conclusion

In summary, we have shown that reduced transcription of topoisomerase IIα associated with doxorubicin treatment is likely to be due to the reduction in Sp1 that has been shown to occur at cell cycle arrest [36] allowing Sp3 to exert its dominant repressive effect. This has important implications for chemotherapy using drugs that target both topoisomerase IIα and cause cell cycle arrest. If the Sp1/Sp3 ratio could be manipulated such that topoisomerase IIα transcription remains at high levels, the drugs classed as topoisomerase II poisons may be more effective. Alternatively, assessment of Sp1/Sp3 ratios in cancer cells may provide an additional prognostic marker for treatment with drugs like doxorubicin that cause cell cycle arrest.

## Methods

### Cell lines

Breast cancer (MDA MB 231) and cervical cancer (HeLa) cells were obtained from ATCC and maintained on minimum essential medium (OPTI-MEM) supplemented with non-essential amino acids (Sigma-Aldrich Pty Ltd., Castle Hill, NSW, Australia), 1% penicillin/streptomycin (Gibco BRL/Invitrogen) and 4% (v/v) fetal calf serum (Gibco BRL/Invitrogen).

### Transient transfection assays

Transient transfection assays were carried out in 12-well tissue culture plates with cells at approximately 60% confluence using 1 μg of reporter vector (topoisomerase IIα-617pGL3Basic [9], 1 μg of pCMV Sport β-galactosidase control vector (Invitrogen) and various amounts of Sp1, and/or Sp3, co-expression vectors using Metafectene (Biontex) transfection reagent according to the manufacturer's instructions. The Sp1 (EF1a/Sp1) co-expression vector was a gift from Dr Merlin Crossley, University of Sydney, Australia) and the Sp3 vector (SPR-2) was from Dr Guntram Suske, Institut fur Molekularbiologie und Tumorforschung, Philipps-Universitat Marburg, Germany. Extracts from cells were harvested 24 hours after transfection and assayed for luciferase activity using a Luciferase Assay Kit (Promega) and FLUOStar galaxy microplate reader (BMG Laboratories) according to the manufacturer's instructions. β-galactosidase was measured using a spectrophotometric assay [37]. Relative luciferase activity was calculated from the ratio between relative light units produced from luciferase assays and the absorbance at 405 nm for the β-galactosidase assays after correction for the appropriate blank values. All values were normalised to the wild type construct that was arbitrarily set at 100%.

### Chromatin immunoprecipitation (ChIP) assays

MDA MB 231 cells were grown for 3 days to approximately 95% confluence in 150 mm tissue culture dishes. The cells were exposed to a single dose of Adriamycin (5 μM) for 1 h [18]. Extractions were carried out at time 0 h (control, unexposed cells) and 2 and 24 hours after exposure.

Cells for each time course were then washed once with PBS (room temperature) and fixed with 1% formaldehyde in PBS (0.14 M NaCl, 2.7 mM KCl, 4.3 mM NaHPO_4_2H_2_O pH 7.2) at 37°C for 10 min, after which they were rinsed twice with ice-cold PBS, scraped, and resuspended in 1 mL of ice-cold PBS. Nuclei were collected by centrifugation at 3000 rpm for 2 minutes at 4°C and the resultant pellet was resuspended in 300 μL of lysis buffer (1% SDS, 5 mM EDTA, 50 mM Tris.HCl, pH 8.1, plus 1× Complete™ mini protease inhibitor [Roche]) then incubated on ice for 10 min. This was followed by sonication (VirSonic Cell Disrupter, VirTis Company) three times at 10% power for 15 sec each to produce fragments in the range of 300–400 bp. After centrifugation the supernatant was diluted (1:10) in buffer (1% Triton X-100, 2 mM EDTA, 150 mM NaCl, 20 mM Tris.HCl, pH 8.1, 1× Complete™ mini protease inhibitor cocktail [Roche]).

Immunoclearing was performed by incubating soluble chromatin with sheared herring sperm DNA (2 mg/ml), 20 μL pre-immune serum and 45 μL of 50% slurry protein A/G plus-agarose beads (Santa Cruz) for 2 h at 4°C. After centrifugation 1 μL (200 mg/mL) of specific antibodies for Sp1 and Sp3 (Santa Cruz) were added to the supernatant and incubated overnight at 4°C. This was followed by the addition of 45 μL protein A/G plus-agarose beads (Sigma), herring sperm DNA and incubation for another 1 h at 4°C. Beads were harvested and washed sequentially for 10 min in 1 mL each of TSE I (0.1% SDS, 1% Triton X-100, 2 mM EDTA, 20 mM Tris.HCl, pH 8.1, 150 mM NaCl), TSE II (0.1% SDS, 1% Triton X-100, 2 mM EDTA, 20 mM Tris.HCl, pH 8.1, 500 mM NaCl), buffer III (0.25 M LiCl, 1% NP-40, 1% deoxycholate, 1 mM EDTA, 10 mM Tris.HCl, pH 8.1), and 1× TE buffer.

The DNA-protein complex was eluted with 100 μL elution buffer (1% SDS, 0.1 M NaHCO_3_) at room temperature for 10 minutes. The eluate was heated at 65°C to reverse formaldehyde cross-links and DNA extracted using a PCR purification kit (QIAGEN) according to the manufacturer's instructions.

### Quantification of ChIP assays

Quantification of ChIP products was carried out with real-time PCR using a LightCycler (Roche) and the LightCycler FastStart DNA Master^PLUS ^SYBR Green kit (Roche). Primers were designed for the GC1 (GCCGTTCATAGGTGGA forward and CGACTAAACAGGCAGGA reverse) and GC2 (TGCTGCGAATACAGACT forward and CTGACGTTGTTAGCGAG reverse) regions in the topoisomerase IIα promoter using LightCycler Probe Design software (Roche) as per the manufacturer's instructions. Real-time PCR protocols were carried out according to the LightCycler manufacturer's instructions. Standard curves for relative quantification were calculated using 1:1, 1:10, 1:100, and 1:1000 dilutions of input sample. The amounts of DNA template for input and immunoprecipitated samples for quantification were equivalent as measured by absorbance at 260 nm. Amplification efficiency was corrected using RelQuant V1.01 software from Roche.

### Protein extraction

Cells were grown to 90% confluence washed in 1× PBS (8 g/L NaCl, 0.2 g/L KCl, 1.15 g/L Na_2_HPO_4_·7H_2_O pH 7.2) and harvested in TEN (40 mM Tris-HCl, pH 7.9, 1 mM EDTA, 0.15 M NaCl) buffer. The cells were lysed in extraction buffer (40 mM Hepes pH 7.9, 0.4 M KCl, 1 mM DTT, 10% glycerol, 1 mM EDTA, and 1× Roche Complete™ mini protease inhibitor) using three freeze-thaw cycles in liquid nitrogen. Whole cell extracts had protein concentrations between 1 and 3 μgmL^-1^.

### Immunoblotting

Protein extracts (~40 μg) were denatured by boiling for five minutes in the presence of SDS and β-mercaptoethanol and then incubated on ice for a further five minutes. The samples were then loaded onto 10% SDS-PAGE mini gels and run in 1× electrophoresis buffer (25 mM Tris, 192 mM Glycine, 0.1% w/v SDS in H_2_O) at 120 V for approximately one hour [38-40]. Proteins were transferred to a positively charged nylon membrane (Roche) in transfer buffer (25 mM Tris, 192 mM glycine in H_2_O) at a constant current of 450 mA for 45 minutes. Immuno-detection was performed with BM Chemiluminescence Blotting Substrate (POD) according to the manufacturer's recommendations (Roche). Membranes were cut into regions representing appropriate molecular weights for each protein and incubated in TBST (0.5 M Tris, 150 mM NaCl, 0.1% v/v Tween 20) including blocking reagent (5% non-fat skim milk in TBST) overnight at 4°C with gentle shaking. Immunodetection was carried out using 1:1000 dilution of Sp1 and Sp3 (Santa Cruz), 1:250 topoisomerase IIα (Santa Cruz), and 1:2000 alpha tubulin (DM 1A) (Sigma) primary antibodies in blocking reagent (2.5% non-fat skim milk in TBST) for three hours at room temperature with shaking.

Membranes were washed three times in TBST for 10 minutes at room temperature. A 1:5000 dilution of horseradish peroxidase conjugated secondary antibody (anti-rabbit, anti-goat or anti-mouse, (Sigma)) was added to 2.5% non-fat skim milk in TBST and incubated for 45 minutes at room temperature. Subsequently, the membranes were washed in TBST three times for 15 min before being immersed in detection solution (99:1 ratio of solution A to B) for two minutes at room temperature. Immunoblots were then detected and quantified using a LAS 1000 Darkbox (Fuji) and Scion Image 4.0.2 (Scion Corporation)

### Chromosome conformation capture (3C) assay

The chromosome conformation capture assay [14] was performed using HeLa cells. Cells were grown to 95% confluence for 3 days and chromatin-crosslinked in buffer (10 mM Tris-Cl pH 7.9, 10 mM MgCl_2_, 50 mM NaCl, 1 mM dithiothreitol) containing 1% formaldehyde for 10 minutes at room temperature. The addition of 0.125 M glycine quenched the reaction before SDS was added to a final concentration of 0.1%. This was followed by removal of non-crosslinked proteins from DNA through incubation at 37°C for 10 minutes. On completion of the incubation, Triton X-100 was added to a final concentration of 1%.

The DNA (5 μL of ~400 ng/μL) was digested with the restriction enzyme *Bsu*36I (Bio-Labs) overnight at 37°C in a final volume of 50 μL. Restriction enzyme inactivation was carried out with the addition of 1.6% SDS and incubation at 65°C for 20 minutes. The reaction was then diluted 15 times and Triton X-100 was added to 1%. The DNA was then ligated for 30 minutes at room temperature using T4 DNA ligase. Crosslinking was reversed by overnight incubation at 65°C in the presence of 5 mg/mL Proteinase K. The ligated (or control) product was then amplified by PCR and detected on a 1.5% agarose gel. The forward (TACTTTTATTTCTTTGAAA) and reverse (ACAAATCAGCGAAAGT) primers used for PCR were specific for the human topoisomerase IIα promoter.

## Authors' contributions

AOW carried out the experimental work and drafted the manuscript. RJI and KMS conceived of the study, suggested the experimental design and critiqued results. KMS constructed the final draft of the manuscript. All authors read and approved the final manuscript.
